# Satisfaction and anxiety level during clinical training among nursing students

**DOI:** 10.1186/s12912-023-01352-3

**Published:** 2023-06-03

**Authors:** Faransa A. Ahmed, Nojoud Alrashidi, Rasha A. Mohamed, Abdulaziz Asiri, Amer Al Ali, Khaled H. Aly, Wael G. Nouh, Nehal A. Demerdash, Salwa Ali Marzouk, Ayat M. Omar, Marzouk M. Marzouk, Safa H. Alkalash, Shimaa M. Moursy, Doaa E. Fadila, Samar S. Eldin, Abeer A. Almowafy

**Affiliations:** 1 College of Applied Medical Sciences in Alnamas, University of Bisha, Bisha, Kingdom of Saudi Arabia; 2 Pediatric Nursing Department, Faculty of Nursing, Assiut University, Assiut, Egypt; 3 Maternal and Child Health, College of Nursing, University of Hail, Hail, Saudi Arabia; 4 Community Health Nursing, Faculty of Nursing, Mansoura University, Mansoura, Egypt; 5 College of Applied Medical Sciences in Bisha, University of Bisha, Bisha, Kingdom of Saudi Arabia; 6 Department of Nursing, College of Applied Medical Sciences, Jouf University, Qurayyat, KSA Saudi Arabia; 7 Maternity and Neonatal Health Nursing, Faculty of Nursing, Fayoum University, Fayoum, Egypt; 8 Public Health and Community Medicine, Damietta Faculty of Medicine, Al-Azhar University, Damietta, Egypt; 9 Community Medicine and Healthcare, Faculty of Medicine, Umm Al-Qura University, Al-Qunfudah, KSA Saudi Arabia; 10 Family Medicine Department, Faculty of Medicine, Menoufia University, Menoufia, Egypt; 11 Gerontological Nursing Department, Faculty of Nursing, Mansoura University, Mansoura, Egypt; 12 Gerontological Nursing, Community Health Nursing, College of Nursing, Taibah University, Madinah, Saudi Arabia; 13 Pediatric Nursing Department, Faculty of Nursing, Menoufia University, Menoufia, Egypt; 14 International Islamic Center for Population Studies and Research, Al-Azhar University, Cairo, Egypt

**Keywords:** Satisfaction, Anxiety, Clinical training, Nursing students

## Abstract

**Background:**

Quality is a primary concern of health care agencies worldwide. A conducive clinical training environment is essential for nursing students to be capable of enhancing their learning experiences and achieving the desired training outcomes.

**Aim:**

This study aimed to examine the satisfaction and anxiety levels during clinical training among nursing students.

**Type of study:**

A descriptive -analytical cross-sectional study design was utilized. The research was conducted at the Faculty of Nursing, Assiut University and Colleges of Applied Medical Sciences in Alnamas and Bisha, University of Bisha. Sampling method: A convenience sampling technique was used. Sample size: a sample of 1052 undergraduate nursing students. The data was gathered via a structured questionnaire including the socio-demographic characteristics and nursing students’ satisfaction with the hospital and laboratory training. Additionally, Self-Rating Anxiety Scale (SAS) was adopted to measure the anxiety level.

**Results:**

The mean age of the studied sample was 21.9 ± 1.83 years, and 56.9% are females. Moreover, 90.1% & 76.4% of the nursing students were satisfied with their hospital and laboratory training. Furthermore, 61.1% & 54.8% of the students had mild levels of anxiety regarding their hospital training and laboratory training, respectively.

**Conclusion:**

The undergraduate nursing students had a high level of satisfaction with their clinical training at the hospitals and laboratories. Moreover, they had mild anxiety related to hospital and laboratory clinical training.

**Recommendations:**

Developing clinical orientation and training programs and improvement strategies to enhance the effectiveness of the clinical training environment. The establishment of a modern, tastefully designed, and fully stocked skill lab for the college's student training should receive more attention.

**Clinical relevance:**

Through the provision of ongoing education about different method of practice, nursing was intended to shape future professional nurses who master core competencies of the profession. Organizations may benefit from developing a comprehensive strategy to achieve an effective teaching program.

## Introduction

Learning premises have been identified as an important factor defining the success of an effective teaching program. The learning environment's atmosphere is a critical component of a successful learning process [1]. Academically, nursing and other related field students learn from classes and clinical teaching environments in order to achieve clinical learning outcomes [2]. One of the critical factors influencing clinical education quality is students' exposure to and preparation for entering the clinical setting [3].

Clinical practice is critical in nursing and medical education because it prepares nursing and medical students to apply what they learn in real theories in clinical practice. It also helps students develop critical thinking skills for problem-solving [4]. Furthermore, it prepares student nurses to be capable of "doing" as well as "knowing" clinical principles in practice [5]. Furthermore, the clinical setting was intended to shape future professional nurses who master core competencies of the profession. This goal is attained through the key factors contributing to successful clinical teaching, including clinical supervision, clear role definition, and a supportive environment that encourages students to engage in active learning [6].

Satisfaction refers to the extent to which students are happy with their learning environment**.** Students' satisfaction as an outcome of the educational process should be of concern to professional education faculties because it has been linked to their later professional attitudes, career commitment, and retention. Teaching faculty should be concerned about students' dissatisfaction with the educational process [7].

According to Fava et al. [8], anxiety is the brain's and body's reaction to all demands. It has both positive and negative effects on one's health and well-being. Anxiety is classified into two categories: state and trait. Individuals' perception of their current situation as threatening and dangerous leads to state anxiety. In general, it is regarded as temporary and common anxiety that everyone experiences [9]. Trait anxiety, on the other hand, is not caused by external threats; rather, it arises from within a person. Trait anxiety is a personality trait unrelated to a person's current situation [10]. Anxiety can also be used to motivate people to perform, such as when studying for an exam [11].

Nursing education is a difficult/stressful educational process because theoretical knowledge and practice are complementary to each other [12]. In nursing, clinical experience has been shown to increase anxiety, which may affect students' training [13]. According to the studies, approximately 15–20% of students have a high level of anxiety, with more than 30% of nursing students having a high level of anxiety. This situation arises as a consequence of the fact that nursing students, unlike students in other professions, face higher pressure as a result of their mistakes causing harm to patients, and their professional lives are threatened. It is well known that low levels of anxiety obligate people to be more careful and strengthen training, whereas high levels of anxiety have a negative impact on clinical training [14].

Additionally, nursing students experience clinical anxiety because many institutes fail to perform well in their students' coping mechanisms, resulting in their students' overall training level remaining low. Thus, it is clear that anxiety has an impact on training, and clinical anxiety, in particular, discourages nursing students [12]. This anxiety may be related to the fact that nursing students must maintain a certain grade point average in order to continue in their highly competitive nursing programs. Furthermore, today's nursing students are frequently non-traditional students who are juggling school and work, and many have their own families [15].

According to the most recent systematic review in the KSA context, nursing students face moderate to high stress during clinical training due to heavy workloads and patient care. According to the reports, nursing students are most stressed while caring for patients, and this is also a period when they are burdened by case studies and theoretical curriculum components [16].

Friendly communication, interpersonal relationships (staff versus students), and capable of supporting students as learners who can contribute to the quality of care can all be signs of a supportive environment [17]. Identifying problems and challenges that these students face in the clinical learning environment can assist stakeholders in resolving these issues and contributing to their professional development as well as their professional survival [3].

Therefore, the knowledge student's satisfaction and anxiety level with clinical training is little identified at the Faculty of Nursing, Assiut University and University of Bisha. In contradiction to this background, this study will examine the satisfaction and anxiety levels during clinical training among the nursing students.

## Methods

### Research aim and questions

This study aimed to examine the satisfaction and anxiety levels during clinical training among nursing students in Assiut University at Egypt and Colleges of Applied Medical Science (Alnamas and Bisha) in University of Bisha at Saudi Arabia. With this in mind, the subsequent study questions were posed:

1. What is the level of nursing students’ satisfaction with clinical training?
2. What is the level of anxiety among students regarding clinical training?
3. If any, what is the relationship between students’ satisfaction/anxiety levels and some of their elected demographic variables?
4. Is there a relationship between student satisfaction and anxiety levels regard to the clinical training?

### Type of study

A descriptive -analytical cross-sectional study was used. This study, on its part, is suitable for obtaining reliable data that make it possible to generate robust conclusions and create new hypotheses that can be investigated with recent research [18].

### Setting

This research was conducted at two faculties of nursing; one in Upper Egypt and the other in the Colleges of Applied Medical Science (Alnamas and Bisha) in University of Bisha at Saudi Arabia (Assiut University and University of Bisha, respectively) during the academic year 2021/2022.

### Sampling method

The study sample was collected using a convenience sampling technique. sample size a sample of 1052 undergraduate nursing students was calculated by G-Power statistical software (version 3.1.9.7; Heinrich-Heine-Universität Düsseldorf, Düsseldorf, Germany). The nursing students enrolled in the third and final grades in the previous mentioned settings were included in this study. Their total number was 1052 students, [644 students from Assiut University at Egypt, and 408 students from Colleges of Applied Medical Science (Alnamas and Bisha) in University of Bisha at Saudi Arabia].

In addition, those who provided written informed approval to join in this research and expressed readiness to answer the questionnaires with the following inclusion criteria: both sexes, students’ age (18–24 years) and practice in laboratory training and hospitals (in-patient-specific units related to the departments of the previously mentioned grades). While, students who had not started clinical rotations, who did not offer written consent to participate in this study, and those who communicated refusal to respond to the questionnaires were not accepted from the current research.

### Study tools

The study tools were supplemented with questions to elicit nursing students' satisfaction and anxiety levels in relation to clinical training either at hospital or laboratory. A closed response was generated from each question in the questionnaires.

#### Tool I

A structured self-administered questionnaire was designed by the researchers after reviewing the related literature [19] to fulfill the aim of the study. The questionnaire was consisted of three parts:

- ▪ **Part 1:** Demographic and academic data about the studied students. It included their age, gender, educational level, department, and residence.
- ▪ **Part 2:** The nursing students’ satisfaction with the hospital training. The questionnaire was consisted of 11 questions with a maximum score of (55).
- ▪ **Part 3:** The nursing students’ satisfaction with the laboratory training at the college. The questionnaire had 11 questions with a maximum score of (55).

Using an ordinal scale, the students were asked to explain their level of agreement with given statements. (1) Very dissatisfied; (2) dissatisfied; (3) unsure; (4) satisfied; (5) very satisfied on a 5-point Likert scale. The satisfaction rate was calculated by multiplying the number of participants who gave positive or negative responses by 100. Using a modified Bloom's criteria cutoff point [20], students' overall satisfaction scores were classified as good if they were between 80 and 100% (44–55), moderate if they were 60–79% (33–43.5), and poor if they were 60% [21].

Content validity of this tool was tested by submitting the tools to a jury of five experts in the field of nursing. The internal consistency of reliability was estimated by the alpha Cronbach's test (values ranging 0,82–0.96), and its result was α = 0.87, and test–retest reliability during a short retest interval was 0.87.

#### Tool II

Self-Rating Anxiety Scale (SAS), compiled by Zung (1971), was adopted to measure the level of anxiety among the study participants [22]. This scale had good psychometric properties, including good internal consistency and concurrent validity. This tool was composed of two parts:-

- ▪ **Part 1:** Assessment of the level of anxiety as regards hospital training. This part was composed of (20 questions) with a maximum score of 80.
- ▪ **Part 2:** Assessment of the level of anxiety as regards laboratory training at the college.This part was composed of (20 questions) with a maximum score of 80.

This SAS scale utilized a 4-point scoring system to assess the frequency of symptoms (1 = no or little time, 2 = a small portion of the time, 3 = a significant amount of time, and 4 = most or all of the time). Fifteen of these use negative words (e.g., I feel more nervous and anxious than usual; I feel afraid for no reason) and were scored on a scale of 1 to 4. The remaining five items were scored in reverse and were used in positive words (e.g., I feel calm and can sit still easily; I can breathe in and out easily). The raw score was calculated by adding the scores of all items, and the standard score was calculated by multiplying the raw score by 1.25. The greater the standard SAS score, the greater the anxiety level.

### Procedure

An official permission was obtained from the dean of the faculty of nursing, from the previous mentioned universities before embarking on the study. After finalizing the study tools, the actual data collection and data analysis was done during the academic year 2021/2022 that took eight months, from October 2021 to May 2022. The questionnaires were administered online using Google questionnaire forms, and the link to the questionnaire was shared to a variety of undergraduate nursing students via various online platforms such as emails and messaging services.

### Pilot study

A pilot study was conducted on (10%) of the study sample (*n* = 100) to check tools' clarity and adequacy. Nursing students recruited in the pilot study were excluded later from the actual study sample. Based on the collected data, the necessary modifications were done, some questions were added, and others were clarified or omitted.

### Ethical considerations

The ethical committee approved the study protocol from Assuit University with code number (IRB no: 3170034). The participants were informed that that they might resign from the study at any time without facing any penalty. Confidentiality and privacy of data was preserved. Electronic informed consent was displayed on the first page of the questionnaire. The names and identification of the students were not collected to protect the confidentiality of the participants. The researchers generated and kept track of their own code numbers.

### Data analysis

Once all necessary information was gathered and verified, it was coded, confirmed, and analyzed using IBM SPSS for Windows software version 25 [23]. Kolmogorov–Smirnov test and the Shapiro–Wilk test were used to test the normality of the data [24]. Statistics were developed to make data easier to understand by presenting it as mean ± standard deviation for quantitative data and calculated frequencies and proportions for nominal and ordinal data. The Chi-square test was used to compare qualitative data among students. Statistical significance was considered at a *P*-value of 0.05, and high statistical significance was considered at a *P*-value of 0.001 across all statistical tests in this study.

## Results

According to the socio-demographic characteristics (age, gender, educational level, department, and residence) of 1052 participating students enrolled in two faculties of nursing [644 students from Assiut University at Egypt, and 408 students from Colleges of Applied Medical Science (Alnamas and Bisha) in University of Bisha at Saudi Arabia] the mean age of the participating students was 21.9 ± 1.83 years, more than half (56.9%) of them were females and almost half (47.5%) recruited from the fourth academic level. The high percentages of the students are unmarried and come from rural area (90.8% and 67.2%, respectively) (Table 1).

**Table 1:** Distribution of the studied sample based on their socio-demographic characteristics (*N* = 1052)

Socio-demographic Characteristics	*N* = 1052	%
**Age (years) Mean ± SD**	21.9 ± 1.83	
▪ 18–20	88	8.4
▪ 21–22	894	85
▪ 22–24	70	6.6
**Sex**		
▪ Female	620	56.9
▪ Male	432	43.1
**Academic year**		
▪ 3^rd^	196	18.6
▪ 4^th^	500	47.5
**University**		
▪ Assiut	644	61.2
▪ Bisha	408	38.8
**Subjects**		
▪ Maternal/Pediatric Nursing	542	51.5
▪ Community health, Mental health and Geriatric Nursing	510	48.5
**Marital status**		
▪ Married	97	9.2
▪ Unmarried	955	90.8
**Residence**		
▪ Urban	345	32.8
▪ Rural area	707	67.2

Table 2 reveals that more than two thirds (64.8% / 63.4% and 70.1% / 60.7%) of the studied students reported that they feel comfortable while performing the evaluation in the hospital and laboratory training and in explaining procedures, medications and therapies respectively. Furthermore, 52% & 49.8% of them mentioned that they feel uncomfortable helping patients and their families through painful procedures in the hospital and laboratory training, respectively. While 65.1% & 59.9% of the studied students expressed that they feel comfortable while supporting patients and their families in times of crisis and grief in the hospital and laboratory training, respectively (Table 2).

**Table 2:** Distribution of students according to their satisfaction regarding hospital and laboratory training with university (Assiutand Bisha) (*N* = 1052)

Variables	Training site	Satisfaction Level		
		**Satisfied** **N(%)**	**Neutral** **N(%)**	**Dissatisfied** **N(%)**
I feel comfortable while performing the evaluation in the training	Hospital	682(64.8)	122(11.6)	248(23.6)
	Laboratory	667(63.4)	46(4.4)	339(32.2)
I feel comfortable in explaining procedures/ medications /therapies	Hospital	737(70.1)	139(13.2)	176(16.7)
	Laboratory	639(60.7)	198(18.8)	215(20.4)
I do not feel comfortable while giving medicines to patients	Hospital	459(43.6)	79(7.5)	514(48.9)
	Laboratory	440(41.8)	55(5.2)	557(52.9)
I feel comfortable doing procedures/ medications/therapies	Hospital	749(71.2)	287(27.3)	16(1.5)
	Laboratory	674(64.1)	125(11.9)	253(24)
I feel uncomfortable helping patients and their families through painful procedures	Hospital	410(39)	95(9)	547(52)
	Laboratory	396(37.6)	132(12.5)	524(49.8)
I feel comfortable while supporting patients and their families in times of crisis and grief	Hospital	685(65.1)	341(32.4)	26(2.5)
	Laboratory	630(59.9)	331(31.5)	91(8.7)
I am concerned about providing nursing care	Hospital	681(64.7)	116(11)	255(24.2)
	Laboratory	656(62.4)	172(16.3)	224(21.3)
I am worried about causing physical harm during this training	Hospital	697(66.3)	49(4.7)	306(29.1)
	Laboratory	668(63.5)	110(10.5)	274(26)
I am concerned about causing psychological harm to dolls during this rotation	Hospital	641(60.9)	37(3.5)	374(35.6)
	Laboratory	609(57.9)	89(8.5)	354(33.7)
I am concerned about causing pain to patients during this rotation	Hospital	673(64)	122(11.6)	257(24.4)
	Laboratory	651(61.9)	129(12.3)	272(25.9)
I worry about dealing with families of patients	Hospital	597(56.7)	85(8.1)	370(35.2)
	Laboratory	576(54.8)	131(12.5)	345(32.8)

According to age, the greatest prevalence of satisfaction in both hospital training and laboratory training (77.5% and 38.9%, respectively) is in the age group of 21–22 years, with a significant difference. On the other side, 1.5% and 1.1% of students above the age of 22 years were dissatisfied with their hospital training and laboratory training compared to the other two older age groups (18–20 years and 21–22 years). With regard to the difference between males and females for the results of satisfaction’s level in the hospital and laboratory training, higher percentages of satisfaction were observed among females (53.2% and 47.3% respectively) compared to males (27.9% and 14.4% respectively) with significant difference (*P* =  > 0.05). In contrast, male students were more dissatisfied (8.4% and 10.5%) in the hospital and laboratory training than females (2.1% and 9.1% respectively).

Another interesting observation was that unmarried students who come from rural areas had a significantly greater frequency of level of satisfaction in the hospital and laboratory training than those married and come from urban areas (*P* =  > 0.05). The same table shows that the percentages of dissatisfaction level in both training hospital and laboratory were significantly high (7.3% and 16.9%, respectively) among students from Assiut University. Moreover, higher percentages of dissatisfaction in hospital and laboratory training are observed among students in the fourth academic level (5.5% and 7.4% respectively) compared to those from the third level (0.6% and 1.2% respectively) without significant differences (Table 3).

**Table 3:** Relation between the studied sample's socio-demographic characteristics and their level of satisfaction in the hospital and laboratory training

Variables	Hospital Training				Laboratory Training			
	**Satisfaction Level**							
	**Satisfied** **N(%)**	**Neutral** **N(%)**	**Dissatisfied** **N(%)**	**Signif. Test**	**Satisfied** **N(%)**	**Neutral** **N(%)**	**Dissatisfied** **N(%)**	**Signif. test**
**Age (years)**								
▪ 18–20	45(4.3)	15(1.4)	28(2.7)	*P* = 0.03* χ^2^ = 255.1	38(3.6)	17(1.6)	33(3.1)	*P* = 0.09 χ^2^ = 8.1
▪ 21–22	815(77.5)	23(2.2)	56(5.3)		409(38.9)	292(27.8)	193(18.3)	
▪ 22–24	44(4.2)	10(1)	16(1.5)		33(3.1)	26(2.5)	11(1.1)	
**Sex**								
▪ Female	560(53.2)	38(3.6)	22(2.1)	*P* < 0.001* χ^2^ = 223.1	498(47.3)	26(2.5)	96(9.1)	*P* = 0.05* χ^2^ = 378
▪ Male	311(29.6)	33(3.1)	88(8.4)		151(14.4)	171(16.3)	110(10.5)	
**Academic year**								
▪ 3^rd^	185(17.6)	5(0.5)	6(0.6)	*P* = 0.08 χ^2^ = 9.7	136(12.9)	47(4.5)	13(1.2)	*P* = 0.07 χ^2^ = 11.3
▪ 4^th^	425(40.4)	17(1.6)	58(5.5)		297(28.2)	125(11.9)	78(7.4)	
**University**								
▪ Assiut	549(52.2)	18(1.7)	77(7.3)	*P* = 0.02* χ^2^ = 377.5	218(20.7)	248(23.6)	178(16.9)	*P* = 0.04** χ^2^ = 497.8
▪ Bisha	388(36.9)	8(0.8)	12(1.1)		267(25.4)	63(6)	78(7.4)	
**Department**								
▪ Maternal/Pediatric Nursing	310(29.5)	142(13.5)	90(8.6)	*P* = 0.03* χ^2^ = 288	297(28.2)	148(14.1)	97(9.2)	*P* < 0.001** χ^2^ = 397.8
▪ Community health, Mental health and Geriatric Nursing	291(27.7)	97(9.2)	122(11.6)		275(26.1)	155(14.7)	80(7.6)	
**Status**								
▪ Married	88(8.4)	7(0.7)	2(0.2)	*P* < 0.001** χ^2^ = 297.3	39(3.7)	55(5.2)	3(0.3)	*P* < 0.001** χ^2^ = 301.6
▪ Unmarried	848(80.6)	23(2.2)	84(8)		678(64.4)	67(6.4)	210(20)	
**Place of Living**								
▪ Urban	311(29.6)	12(1.1)	22(2.1)	*P* = 0.01* χ^2^ = 347.8	195(18.5)	91(8.7)	59(5.6)	*P* = 0.03* χ^2^ = 223.1
▪ Rural area	586(55.7)	33(3.1)	88(8.4)		305(29)	293(27.9)	109(10.4)	

χ^2^: Chi-square test

P: Significance

^*^Significant (*P* < 0.05)

^**^Highly significant (*P* < 0.01)

Table 4 reveals that no significant differences between students’ age groups or academic level and the level of anxiety in hospital training (*P* > 0.05) while in laboratory training severe anxiety is significantly more prevalent among students in the age group of 21–22 years ( *P* = 0.05). Moreover, the odds of severe anxiety in the hospital and laboratory training were significantly high among the females (*P* = 0.02 and *P* = 0.01, respectively), those who come from rural areas (*P* = 0.001 and *P* = 0.02, respectively) and in Assiut University (*P* = 0.03 and *P* = 0.002, respectively) (Table 4).

**Table 4:** Relationship between the studied sample's socio-demographic characteristics and their level of anxiety in the hospital and laboratory training

Variables	Hospital Training				Laboratory Training			
	**Anxiety Level**							
	**Mild** **N(%)**	**Moderate** **N(%)**	**Severe** **N(%)**	**Signif. test**	**Mild** **N(%)**	**Moderate** **N(%)**	**Severe** **N(%)**	**Signif. Test**
**Age (years)**								
▪ 18–20	11(1)	51(4.8)	26(2.5)	*P* = 0.09 χ^2^ = 7.5	24(2.3)	27(2.6)	37(3.5)	*P* = 0.05* χ^2^ = 215.1
▪ 21–22	278(26.4)	289(27.5)	327(31.1)		276(26.2)	284(27)	334(31.7)	
▪ 22–24	19(1.8)	23(2.2)	28(2.7)		18(1.7)	23(2.2)	29(2.8)	
**Sex**								
▪ Female	122(11.6)	157(14.9)	341(32.4)	*P* = 0.02* χ^2^ = 278.3	205(19.5)	140(13.3)	275(26.1)	*P* = 0.01* χ^2^ = 254
▪ Male	17(1.6)	147(14)	268(25.5)		55(5.2)	167(15.9)	210(20)	
**Academic Level**								
▪ 3^rd^	27(2.6)	37(3.5)	132(12.5)	*P* = 0.09 χ^2^ = 8.9	51(4.8)	43(4.1)	102(9.7)	*P* = 0.06 χ^2^ = 11.1
▪ 4^th^	56(5.3)	143(13.6)	301(28.6)		148(14.1)	164(15.6)	188(17.9)	
**University**								
▪ Assiut	109(10.4)	212(20.2)	323(30.7)	*P* = 0.03* χ^2^ = 360	87(8.3)	213(20.2)	344(32.7)	*P* = 0.002* χ^2^ = 420.2
▪ Bisha	96(9.1)	105(10)	207(19.7)		141(13.4)	63(6)	204(19.4)	
**Department**								
▪ Maternal/Pediatric Nursing	110(10.5)	178(16.9)	254(24.1)	*P* = 0.07 χ^2^ = 7.9	101(9.6)	131(12.5)	310(29.5)	*P* = 0.03* χ^2^ = 286.6
▪ Community health, Mental health and Geriatric Nursing	138(13.1)	166(15.8)	206(19.6)		33(3.1)	198(18.8)	279(26.5)	
**Status**								
▪ Married	5(0.5)	16(1.5)	76(7.2)	*P* = 0.02* χ^2^ = 370.1	8(0.8)	13(1.2)	76(7.2)	*P* = 0.07 χ^2^ = 5.1
▪ Unmarried	187(17.8)	295(28)	473(45)		232(22.1)	256(24.3)	467(44.4)	
**Place of Living**								
▪ Urban	89(8.5)	97(9.2)	159(15.2)	*P* < 0.001** χ^2^ = 387.7	131(12.5)	69(6.6)	145(13.8)	*P* = 0.02* χ^2^ = 430.8
▪ Rural area	17(1.6)	213(20.2)	477(45.3)		27(2.6)	209(19.9)	471(44.8)	

χ^2^: Chi-square test

P: Significance

^*^Significant (*P* < 0.05)

^**^Highly significant (*P* < 0.01)

**Fig. 1:**
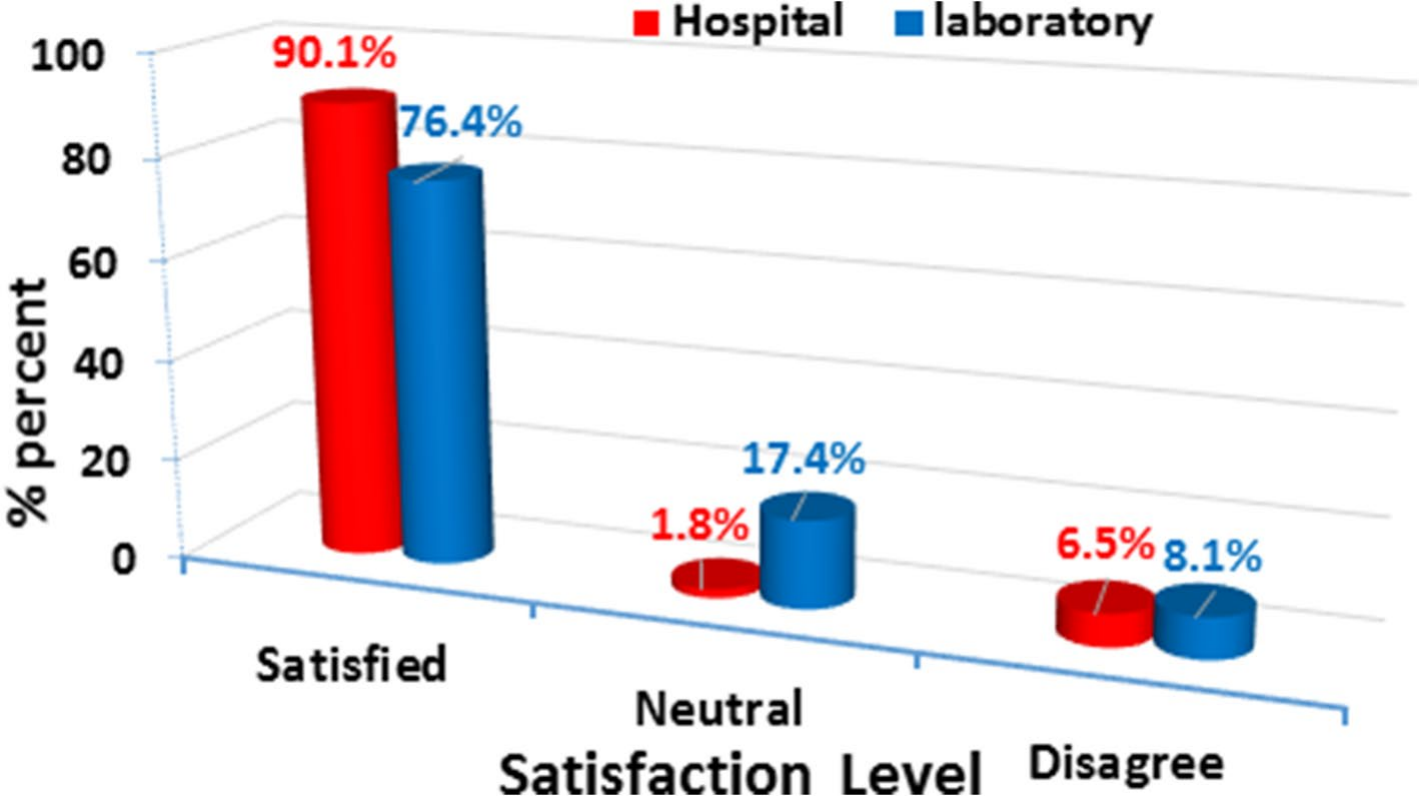
Satisfaction Level of the Studied Students Based on Hospital and Laboratory Training

Figure 1 illustrates the overall students' satisfaction level in the hospital and laboratory training. that most of the students (90.1%)were satisfied regarding their hospital training compared with 6.5% who were not satisfied. However, the majority of the students (76.4%) were satisfied with their laboratory training compared with 8.1% who were not satisfied.

**Fig. 2:**
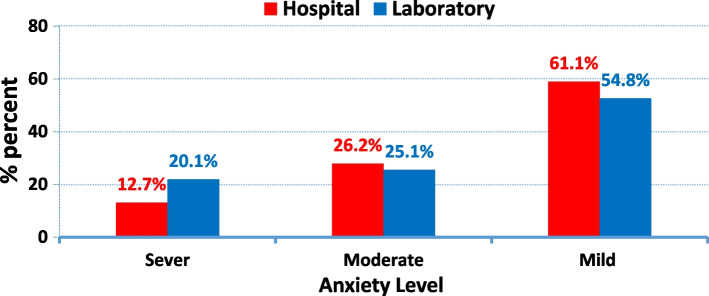
Anxiety Level of the Studied Students Based on Hospital and Laboratory Training

Figure 2 denotes that more than half of the students had mild anxiety level in their hospital training and laboratory training (61.1% and 54.8%, respectively). While the percentage of sever anxiety level was higher in laboratory training than hospital training (20.1% and 12.7%, respectively).

## Discussion

This study investigated satisfaction and anxiety levels during clinical training among nursing students. at two faculty of nursing. Clinical training environments are areas of clinical education where undergraduate students can improve clinical application skills [25]. Many challenges, difficulties, and overwhelming work, such as student tension and anxiety during clinical training, are part of the basic framework for a clinical training environment [26]. The current study's demographic findings revealed that mean age of the studied sample was 21.9 ± 1.83 years, more than half of them were females, and almost half were recruited from the fourth academic level. A similar study on depression, anxiety, and stress among undergraduate nursing students at a Sri Lankan public university found 30.4% of males and 69.6% of females aged 21 to 27. Furthermore, the vast majority of respondents were fourth-year students [13].

Satisfaction could be used as the main factor in the development of clinical training environments in order to meet students' needs and expectations [27]. According to the current study findings, over three quarter of the students were satisfied with their hospital and laboratory training. It appears that involving students in patient care improves the training environment because students learn through role modelling and effective supervision. In line with this finding, Ibrahim et al. (2019) discovered at Alexandria University in Egypt that the undergraduate nursing students have a high level of satisfaction with the clinical training environment regarding all components of the clinical placement [28]. Likewise, these findings are consistent with the findings of previous studies [7, 29].

Disaggregated data revealed that sociodemographic characteristics are associated with satisfaction level. In relation to gender, the current research showed that female students had a higher level of satisfaction in both laboratory and hospital training than males reported, whereas dissatisfaction is more prevalent in male students (*P* < 0.001). This is in agreement with Alatawi et al.'s (2020) findings [25].

With regard to the academic level, fourth-grade students reported the highest level of dissatisfaction when compared to previous grade. This finding could be explained by the fact that the training objectives and activities differed in terms of academic progress. This is relatively close to Wang et al., 2019, who reported that the first-year students were the most satisfied than the last year. Furthermore, they stated that the satisfaction decreases as students progressed through the program. Inversely, Brynildsen et al., 2014 [30] concluded that first-year students experienced high levels of physical and mental stress due to their limited capacities in terms of basic clinical skills.

Nursing teaching is practiced differently across countries. Hence, it is beneficial to understand the factors that influence students' satisfaction levels. This study explored that satisfaction in both training hospital and laboratory are significantly lower among students at Assiut university. The students in the Saudi Arabia have a much higher satisfaction level. Different characteristics of the nursing students and difference in the curriculum may explain this finding. That the opportunities to practice different tasks are facilitating factors for students' nursing practice. The actual tasks assigned and patient care experience might be major factors determining nursing satisfaction levels [31].

Nursing is considered to be one of the most stressful and emotionally demanding fields which results from the gap between theory-based learning and experiences in clinical practice [32]. The current research found that more than half of the students experienced mild anxiety during their hospital and laboratory training. These findings are congruent with a study conducted by Rodrigues Lavina et al. [21] which showed that 83% of the students had a normal range of anxiety, while the remaining students had a moderate level of anxiety. Additionally, a similar observation noted by [33, 34]. Furthermore, a study conducted in KSA revealed that stress levels among nursing students during their clinical training were moderate due to various stressors [35]. According to the current study results, the other two level of the anxiety (moderate and sever) are still high. This finding was reinforced by previous study, which stated that the clinical training is a stressful aspect of nursing students [36].

Socio demographic determinants of nursing students' anxiety levels were further investigated in the study. Awotrhy note is the observed gender difference. The current study findings explored that highly significant relationship between the level of anxiety and the student gender. Female students were more anxious than male students whether in hospital training or in the laboratory. This is consistent with Wedgeworth (2016), who conducted a study on nursing students' anxiety in a clinical setting and reported a significant relationship between the level of anxiety and participants' gender. This can be explained by the fact that females are more sensitive to emotions than males, making them more prone to anxiety. This is in an agreement with a study involving students in healthcare professions, which showed that females had a higher percentage of high anxiety than males [14]. On the other side, this finding contradicts those reported by Otim et al. (2021) [10]. In contrast to the current study findings, a study by Rodrigues Lavina et al. (2021) found no link between anxiety and gender [21, 37].

Across the studied counries, nursing students who are Egyptian and/or resided rural areas reported significantly higher levels of stress as compared to students lives in Saudi Arabia and/or urban regions. This could be attributed to difference in the exposure to type of stressors according to the origin. This finding may be also explained by differences in the individual country's nursing curriculum. In addition to differences in social and economic situations and possibly cultural differences [38]. In agreement, Persike and Seiffge-Krenke, who assumed that prevailing value systems and cultural norms determine the way of responding to stress [39].

## Limitation of the study

The findings of this study have limitations because they are based on student self-reported data. As a result, there is a possibility that reporting bias occurred as a result of how respondents interpreted the questions, their desire to express their emotions in a particular way, or simply because of inaccurate responses. However, the study was conducted at just two colleges, which limits its generalizability to other academic settings.

## Conclusion

According to the current study findings, the undergraduate nursing students at the Faculty of Nursing, Assiut University and the Colleges of Applied Medical Sciences, University of Bisha, have a high level of satisfaction with their clinical training at the hospitals and laboratories. As well they have a mild anxiety level related to hospital and laboratory clinical training. Further, there is a highly significant relationship between the satisfaction level of laboratory and hospital training and students' gender and academic level. While clinical practice provided the students with the opportunity to gain knowledge and develop skills in the preparation of caring for clients, the present findings revealed that the participants perceived it to be stressful and anxiety-producing.

### Recommendations

Considering the study's findings, the researchers have made a few recommendations for future research. First and foremost, there is an elevating need to enhance the clinical orientation and training programs through developing training programs and improvement strategies to enhance the effectiveness of the clinical training environment for increasing students' satisfaction and reducing anxiety levels.

The establishment of a modern, tastefully designed, and fully stocked skill lab for the college's student training should receive more attention. Besides, providing orientation and counselling programs on campus to raise awareness and provide academic support to anxious students is also an imperative factor. As a result, the collaboration between nursing universities and teaching hospitals is of paramount significance in order to optimize the clinical training environment. Lastly, educational institutions and all other stakeholders must work together to eliminate the numerous challenges students face in the clinical setting.

### Implication for practice

The findings of this study could aid educators in identifying nursing students’ requirements, enabling their education in the appropriate clinical environment, and initiating effective strategies to cope with stressors which subsequently improve patients’ care.

## Data Availability

The datasets generated during and/or analyzed during the current study are available from the corresponding author on reasonable request.
